# Supplementary material for: Modeling glioblastoma heterogeneity as a dynamic network of cell states
Source: Mol Syst Biol. 2021 Sep 16;17(9):e10105. doi: 10.15252/msb.202010105 (PMC8444284; doi:10.15252/msb.202010105)
Supplement: Supplementary file 6 — Source Data for Figure 5 [file MSB-17-e10105-s004.zip › Figure5A_sourcedata/GSEA_3071/nosigdb_stateIV.GseaPreranked.1622795164626/LARSSON_STATE_6.html]

Details for gene set LARSSON\_STATE\_6[GSEA]

|  || Dataset | state53071 |
| Phenotype | NoPhenotypeAvailable |
| Upregulated in class | na\_pos |
| GeneSet | LARSSON\_STATE\_6 |
| Enrichment Score (ES) | 0.636169 |
| Normalized Enrichment Score (NES) | 3.307431 |
| Nominal p-value | 0.0 |
| FDR q-value | 0.0 |
| FWER p-Value | 0.0 |
Table: GSEA Results Summary

  

Fig 1: Enrichment plot: LARSSON\_STATE\_6      
 Profile of the Running ES Score & Positions of GeneSet Members on the Rank Ordered List

  

| PROBE | GENE SYMBOL | GENE\_TITLE | RANK IN GENE LIST | RANK METRIC SCORE | RUNNING ES | CORE ENRICHMENT || 1 | GFAP |  |  | 6 | 2.698 | 0.0402 | Yes |
| 2 | PMP2 |  |  | 11 | 2.494 | 0.0777 | Yes |
| 3 | S100B |  |  | 12 | 2.461 | 0.1154 | Yes |
| 4 | FABP7 |  |  | 14 | 2.398 | 0.1520 | Yes |
| 5 | CRYAB |  |  | 15 | 2.373 | 0.1884 | Yes |
| 6 | BEX1 |  |  | 18 | 2.252 | 0.2225 | Yes |
| 7 | CLU |  |  | 27 | 2.064 | 0.2527 | Yes |
| 8 | TUBB2B |  |  | 69 | 1.731 | 0.2714 | Yes |
| 9 | ZNF428 |  |  | 101 | 1.525 | 0.2888 | Yes |
| 10 | NFIB |  |  | 117 | 1.491 | 0.3088 | Yes |
| 11 | ID2 |  |  | 149 | 1.378 | 0.3240 | Yes |
| 12 | TUBA1A |  |  | 165 | 1.337 | 0.3416 | Yes |
| 13 | RND2 |  |  | 182 | 1.298 | 0.3584 | Yes |
| 14 | C1QL1 |  |  | 195 | 1.272 | 0.3756 | Yes |
| 15 | SOX9 |  |  | 214 | 1.223 | 0.3909 | Yes |
| 16 | CTXN1 |  |  | 226 | 1.192 | 0.4071 | Yes |
| 17 | FABP5 |  |  | 230 | 1.187 | 0.4247 | Yes |
| 18 | TNFRSF21 |  |  | 232 | 1.184 | 0.4427 | Yes |
| 19 | PFN2 |  |  | 270 | 1.107 | 0.4525 | Yes |
| 20 | FOXO3 |  |  | 291 | 1.054 | 0.4649 | Yes |
| 21 | PBX1 |  |  | 332 | 0.987 | 0.4723 | Yes |
| 22 | GNAI2 |  |  | 340 | 0.980 | 0.4860 | Yes |
| 23 | FEZ1 |  |  | 365 | 0.949 | 0.4960 | Yes |
| 24 | SOX4 |  |  | 377 | 0.928 | 0.5081 | Yes |
| 25 | SESN3 |  |  | 380 | 0.925 | 0.5219 | Yes |
| 26 | MARCKSL1 |  |  | 417 | 0.888 | 0.5286 | Yes |
| 27 | OLFM2 |  |  | 461 | 0.847 | 0.5333 | Yes |
| 28 | PNRC1 |  |  | 481 | 0.830 | 0.5424 | Yes |
| 29 | MXD4 |  |  | 526 | 0.793 | 0.5461 | Yes |
| 30 | METRN |  |  | 531 | 0.783 | 0.5574 | Yes |
| 31 | PTMS |  |  | 549 | 0.763 | 0.5658 | Yes |
| 32 | MARCH2 |  |  | 556 | 0.759 | 0.5763 | Yes |
| 33 | GABARAPL2 |  |  | 583 | 0.739 | 0.5827 | Yes |
| 34 | C1orf122 |  |  | 635 | 0.706 | 0.5837 | Yes |
| 35 | C9orf16 |  |  | 641 | 0.701 | 0.5935 | Yes |
| 36 | PLEKHB1 |  |  | 648 | 0.692 | 0.6029 | Yes |
| 37 | BEX3 |  |  | 707 | 0.655 | 0.6019 | Yes |
| 38 | RTL8C |  |  | 715 | 0.650 | 0.6105 | Yes |
| 39 | CITED1 |  |  | 736 | 0.639 | 0.6165 | Yes |
| 40 | COL4A3BP |  |  | 825 | 0.590 | 0.6086 | Yes |
| 41 | SAT1 |  |  | 859 | 0.575 | 0.6111 | Yes |
| 42 | RAB31 |  |  | 864 | 0.574 | 0.6192 | Yes |
| 43 | FAM20C |  |  | 873 | 0.571 | 0.6264 | Yes |
| 44 | CTNNA1 |  |  | 909 | 0.557 | 0.6282 | Yes |
| 45 | MT3 |  |  | 913 | 0.555 | 0.6362 | Yes |
| 46 | ZMAT3 |  |  | 966 | 0.536 | 0.6344 | No |
| 47 | SH3BGRL |  |  | 1001 | 0.522 | 0.6359 | No |
| 48 | QKI |  |  | 1078 | 0.500 | 0.6290 | No |
| 49 | RPS11 |  |  | 1100 | 0.492 | 0.6325 | No |
| 50 | TSC22D4 |  |  | 1176 | 0.472 | 0.6254 | No |
| 51 | IRS2 |  |  | 1218 | 0.458 | 0.6246 | No |
| 52 | SNX3 |  |  | 1264 | 0.450 | 0.6229 | No |
| 53 | MAP1LC3A |  |  | 1313 | 0.438 | 0.6204 | No |
| 54 | TUBB2A |  |  | 1373 | 0.422 | 0.6155 | No |
| 55 | FYN |  |  | 1483 | 0.394 | 0.6007 | No |
| 56 | FKBP8 |  |  | 1513 | 0.386 | 0.6010 | No |
| 57 | TOMM7 |  |  | 1686 | 0.353 | 0.5735 | No |
| 58 | MARCKS |  |  | 1713 | 0.348 | 0.5738 | No |
| 59 | SMDT1 |  |  | 1718 | 0.347 | 0.5784 | No |
| 60 | LAMTOR4 |  |  | 2075 | 0.287 | 0.5146 | No |
| 61 | ATP6V1G1 |  |  | 2148 | 0.277 | 0.5050 | No |
| 62 | SKP1 |  |  | 2150 | 0.276 | 0.5090 | No |
| 63 | SCP2 |  |  | 2368 | -0.255 | 0.4714 | No |
| 64 | TPT1 |  |  | 2649 | -0.290 | 0.4221 | No |
| 65 | SEPT7 |  |  | 2703 | -0.295 | 0.4165 | No |
| 66 | EFTUD2 |  |  | 2784 | -0.304 | 0.4058 | No |
| 67 | TXNIP |  |  | 2887 | -0.317 | 0.3911 | No |
| 68 | ID1 |  |  | 3973 | -0.504 | 0.1909 | No |
| 69 | FTL |  |  | 4350 | -0.615 | 0.1283 | No |
| 70 | LGALS3 |  |  | 4418 | -0.641 | 0.1253 | No |
| 71 | C4orf48 |  |  | 4536 | -0.698 | 0.1136 | No |
| 72 | ID3 |  |  | 4686 | -0.788 | 0.0971 | No |
| 73 | HOPX |  |  | 5056 | -1.204 | 0.0448 | No |
Table: GSEA details [plain text format]

  

Fig 2: LARSSON\_STATE\_6: Random ES distribution      
 Gene set null distribution of ES for **LARSSON\_STATE\_6**
